# Metabolomic signature associated with reproduction-regulated aging in *Caenorhabditis elegans*

**DOI:** 10.18632/aging.101170

**Published:** 2017-02-06

**Authors:** Qin-Li Wan, Xiaohuo Shi, Jiangxin Liu, Ai-Jun Ding, Yuan-Zhu Pu, Zhigang Li, Gui-Sheng Wu, Huai-Rong Luo

**Affiliations:** ^1^ State Key Laboratory of Phytochemistry and Plant Resources in West China, Kunming Institute of Botany, Chinese Academy of Sciences, Kunming, Yunnan 650201, China; ^2^ University of Chinese Academy of Sciences, Beijing 100039, China; ^3^ Yunnan Key Laboratory of Natural Medicinal Chemistry, Kunming, Yunnan 650201, China; ^4^ Shineway Pharmaceutical Co., Ltd, Sanhe, Hebei 065201, China; ^5^ Key Laboratory for Aging and Regenerative Medicine, Department of Pharmacology, School of Pharmacy, Southwest Medical University, Luzhou, Sichuan 646000, China

**Keywords:** Caenorhabditis elegans, aging, reproduction, metabolome, UPLC-MS, NMR

## Abstract

In *Caenorhabditis elegans (C. elegans)*, ablation of germline stem cells (GSCs) leads to infertility, which extends lifespan. It has been reported that aging and reproduction are both inextricably associated with metabolism. However, few studies have investigated the roles of polar small molecules metabolism in regulating longevity by reproduction. In this work, we combined the nuclear magnetic resonance (NMR) and ultra-performance liquid chromatography-mass spectrometry (UPLC-MS) to profile the water-soluble metabolome in *C. elegans*. Comparing the metabolic fingerprint between two physiological ages among different mutants, our results demonstrate that aging is characterized by metabolome remodeling and metabolic decline. In addition, by analyzing the metabolic profiles of long-lived germline-less *glp-1* mutants, we discovered that *glp-1* mutants regulate the levels of many age-variant metabolites to attenuate aging, including elevated concentrations of the pyrimidine and purine metabolism intermediates and decreased concentrations of the citric acid cycle intermediates. Interestingly, by analyzing the metabolome of *daf-16;glp-1* double mutants, our results revealed that some metabolic exchange contributing to germline-mediated longevity was mediated by transcription factor FOXO/DAF-16, including pyrimidine metabolism and the TCA cycle. Based on a comprehensive metabolic analysis, we provide novel insight into the relationship between longevity and metabolism regulated by germline signals in *C. elegans*

## INTRODUCTION

Aging is an inevitable and complex part of life and has been a fascinating phenomenon for several thousands of years. Reproductive capacity is closely related to aging, and previous studies have demonstrated the apparent trade-off between reproduction and aging. Consistent with this idea, longevity can be achieved by sacrificing fertile potential in many species, including *Caenorhab-ditis elegans (C. elegans)* [1, 2], drosophila melanogaster [3], and humans [4], suggesting that the relationship between reproduction and aging is conserved.

Reproduction is an energetically costly process, and it has been reported that reduced reproduction is associated with elevated fat storage and prolonged lifespans in multiple organisms [5, 6]. These findings seem to indicate that the longevity of a species is a direct result how it distributes its resources between reproduction and survival. In the nematode *C. elegans*, laser ablation of germline stem cells (GSCs) precursors or genetic ablation of GLP-1/Notch signaling causes GSC proliferation to be inhibited, which can activate signals in somatic tissues that significantly lengthen lifespan [1] and alter lipid metabolism [7], known as the Glp (germ-line proliferation defective) phenotype. Moreover, researchers have begun to reveal the molecular mechanisms by which signals from the reproductive system influence lipid metabolism and lifespan [8, 9].

In *C. elegans*, during germline quiescence, steroidal signaling (DA/DAF-12), microRNA mir-7, and ankyrin repeat-containing protein KRI-1 regulate and prompt the nuclear localization and activation of DAF-16 [1, 10–12]. In addition, DAF-16 nuclear activity is regulated by assuming complexes comprised of FTT-2, PHI-62, and TCER-1. These proteins collaborate in transcriptional complexes to regulate germline signals to extend lifespan [13–16]. In addition, in germline-less worms, SKN-1 was also regulated in parallel with DAF-12 and DAF-16 [17]. Additionally, NHR-80 transcriptional complexes are also regulated by inputs from the germline perhaps in a manner independent of DAF-16 but partly dependent on DAF-12 [18]. Germline loss also stimulated TOR downregulation, which in turn, upregulating PHA-4 and autophagy processes [19]. Altogether, during germline defect, the different transcription factors and nuclear receptors function in a complex and sophisticated network, which initiates a cascade of dramatic events, including autophagy, fatty acid lipolysis, stress resistance and other processes, to enhance homeostasis and increase lifespan.

Compared to the detailed studies on germline signals regulating lipid metabolism and longevity in *C. elegans*, it remains unknown whether or how other small molecules, including amino acids and sugar, play roles in germline-mediated longevity.

Recent studies have demonstrated that the use of high-throughput ‘-omic’ approaches can increase our understanding of the global variation that accompanies aging and anti-aging [20]. Metabolomics, the untargeted profiling of metabolites, is the real endpoint of physiological regulatory processes, which aims to identify perturbations in biochemical networks and to further extend our understanding of the molecular mechanisms underlying specific biological function in complex organisms [21–23]. By monitoring many related and unrelated small molecules, the metabolic profile can provide a snapshot of highly complex metabolic exchanges, which integrate information from multiple levels of organization, including the genome, the transcriptome, the proteome, the environment and their interactions [24]. Furthermore, metabolomics have been used in aging studies of *C. elegans*. For example, Fuchs and co-workers found the existence of a common metabolic signature for extending lifespan by investigating metabolic changes of the long-lived worm mutants using high-resolution ^1^H NMR spectroscopy [25]. Another study that utilized a high-resolution ^1^H NMR metabolomics approach found that, compared with wild type, higher concentrations of branched-chain amino acids (BCAA) were detected in long-lived mutants [26]. Additionally, a previous study also reported metabolic variation associated with aging and specially found that low phosphocholine (PCho) was correlated with high life expectancy based on a ^1^H high-resolution magic-angle spinning (HR-MAS) nuclear magnetic resonance (NMR) analysis of intact worms [27]. Other researchers have used a GC-MS meta-bolomics approach to suggest that a-ketoacids and a-hydroxyacids were related to extend lifespans of long-lived mitochondrial mutants but not of other long-lived mutants or short-lived mutants [28]. Furthermore, questions have been raised as to whether we can investigate the relationship of reproduction and longevity by using a comprehensive metabolomics approach.

In this work, we used *C. elegans* hermaphrodites as a model system to investigate how metabolic pathways changed during aging and how germline signals regulate metabolism to attenuate aging. Here, we assessed the metabolic phenotype of whole *C. elegans* animals by combining NMR and UPLC-MS. The effect of germline signals on aging-related metabolism was analyzed using multivariate statistics, including unsupervised principal component analysis (PCA), hierarchical and supervised orthogonal projection to latent structure with discriminant analysis (OPLS-DA). By characterizing the metabolic profile of wild-type, *glp-1* mutants, and *daf-16;glp-1* double mutants, our results demonstrate that *glp-1* mutants regulate some age-related metabolic variations to achieve a long-lived phenotype, and some metabolic pathways influenced by germline-less signals are mediated by FOXO/DAF-16.

## RESULTS

### The metabolomics analysis associated with aging in *C. elegans*

**Figure 1:**
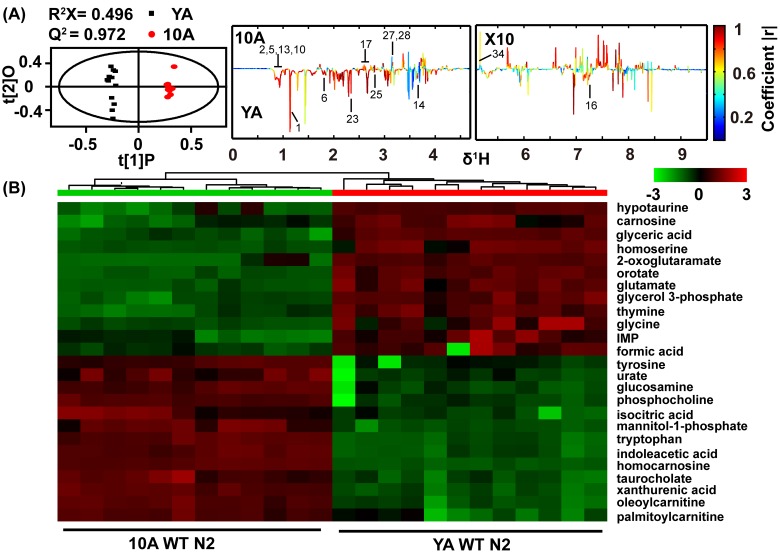
Age-related comprehensive metabolomics analysis in wide type *C. elegans* **(A)** Scores and loading plots from OPLS-DA model of NMR data for YA (young adult) and 10A (10 days of adulthood) wild-type N2. The region of δ5.0-9.5 in the loading plot was vertically expanded 10 times. NMR metabolites assignment showed in the Table S1 (Supplemental information). **(B)** Metabolomics analysis from UPLC-MS data for YA and 10A wild-type N2. Heatmap plot showed that 25 most importantly different metabolites from the samples according to their aging status. More differences metabolites were listed in the Table S2 (Supplemental information). Data are presented using hierarchical clustering (Pearson's correlation coefficient). Metabolite abundance level were reflected in the heat-maps using colors, and with blue being lower and red higher when comparing the mean metabolite abundance value. Using the distance function 1-correlation in hierarchical clustering determine the order of metabolite and animal.

Aging populations may accumulate novel metabolites or abnormal levels of normal metabolites [27, 29]. To characterize the metabolic changes that occur during aging in *C. elegans*, we acquired ^1^H NMR metabolic profiles of wild-type N2 in young adults (YA) (egg-laying has not commenced) and day 10 adults (10A) (aging, egg-laying has ceased) and analyzed the data using two multivariate statistical methods: unsupervised PCA [30] and supervised OPLS-DA [31] models (Figure 1A and Figure 6A). Because the stochastic component of aging in *C. elegans* is particularly obvious after day 10, we did not consider time points after this time [10]. Importantly, albeit wild-type worms can live for up to 4 weeks, many aging-related phenotypes, such as decreased rates of pharyngeal pumping, muscle deterioration and mitochondrial fission, are evident at day 10 [1, 11]. Moreover, by choosing these two time points, the effect of egg-laying on metabolism was also diminished.

Our results showed that, compared with YA N2, the concentrations of alanine, cystathionine, isoleucine, leucine, lysine, phenylalanine, glycine, valine, 3-aminoisobutyric acid, and succinate were decreased in the 10A N2 worms, while those of GPC, phospho-rylcholine, aspartate and trehalose were increased (Figure 1A). The YA and 10A worms were virtually isogenic and maintained in a constant environment, and therefore, any bias linked to individual phenotype was excluded by our sampling conditions. Accordingly, these data reveal that the metabolic profiles related to the chronological age of *C. elegans* and may provide a characteristic fingerprint that is linked to physiological aging.

The overlapping signals of the one-dimensional NMR metabolomics limited the quantitative analysis of the metabolites [32]. To expand upon the characterization of metabolites in aging worms and to identify possible novel aging biomarkers, we further analyzed the metabolic signature during aging using UPLC-MS. The different metabolites between 10A and YA N2 worms determined by UPLC-MS were in good agreement with NMR. Additionally, the UPLC-MS analysis identified more distinct metabolites, such as decreased levels of purine and pyrimidine and increased levels of taurocholate in aged worms. Figure 1B listed the top 25 different metabolites from the UPLC-MS data for 10A worms compared with YA worms, and additional altered metabolites were summarized in Table S2 (supplemental information).

### The progression of aging resulted from metabolome remodeling

Age-related remodeling has previously been described for the epigenome, the transcriptome, and the proteome [12–14]. Our work offers insight into aging through a comprehensive assessment of the variations of many endogenous polar small molecules in *C. elegans*. We questioned whether subsets of metabolites connected to a particular characteristic or characteristic combination were enriched for specific metabolic pathways.

**Figure 2:**
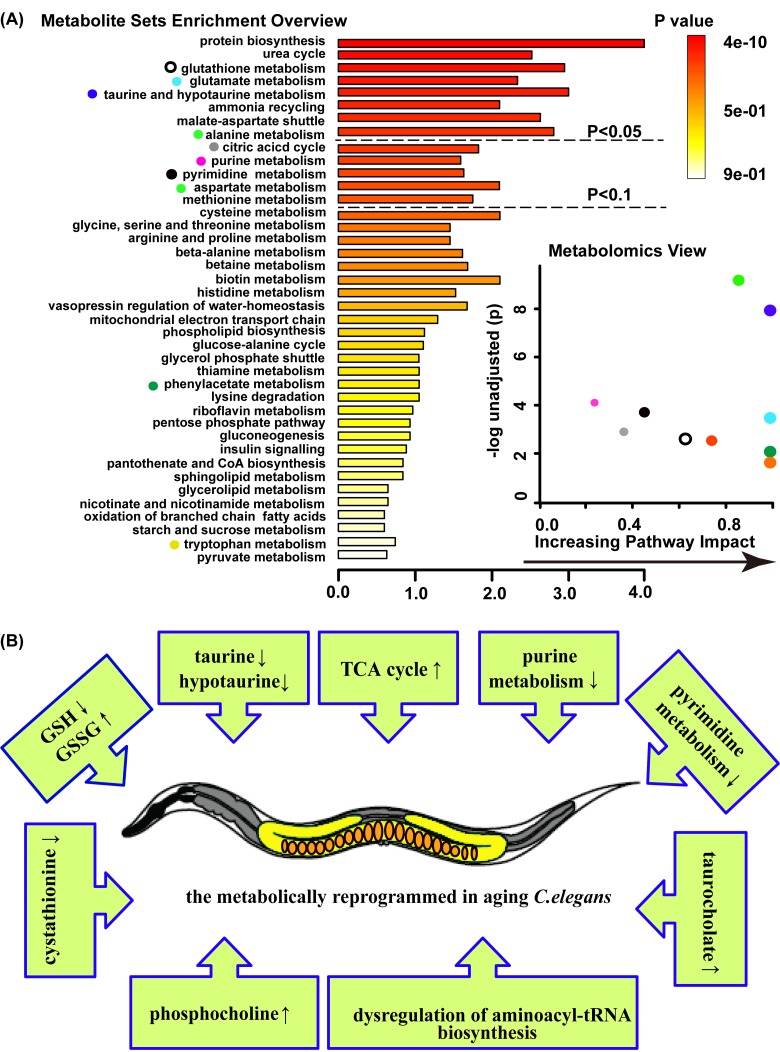
Age-related metabolic remodeling in *C. elegans* (**A)** Summary plot for metabolite enrichment analysis(MSEA) (left panel), where metabolite sets were ranked according to Holm p-value, and the cut off of Holm p-value showed with hatched lines (the panel overviews metabolites repeated measured by Mann-Whitney U test, *p*<0.05). Metabolomics view (right panel) reflects key nodes in metabolic pathways that have been significantly altered with aging, and in which x-axis reflects the increasing metabolic pathway impact according to the between centrality measure. MSEA was performed using package global test and the metabolome view displayed the pathway topological analysis. (**B)** Model on the aging *C. elegan*s response entails a complex series of metabolic change.

Among metabolites that change with age, through a metabolite enrichment analysis (MSEA [33]) and a metabolome view analysis, we identified several groups that strongly enriched for certain pathways and of specific interest from an aging perspective, including glutathione metabolism, taurine and hypotaurine metabolism, purine metabolism, pyrimidine metabolism, citric acid cycle (TCA cycle), aminoacyl-tRNA biosynthesis, glycerophospholipid metabolism, starch and sucrose metabolism and glutamate metabolism (Figure 2A and B).

Among them, the glutathione metabolism includes GSH and GSSG. In aged worms, the level of glutathione (GSH) significantly decreased, while oxidized glutathione (GSSG) increased. The second group of molecules related to aging included taurine and hypotaurine, which are antioxidants and significantly declined with age. Moreover, phosphocholine, the intermediate metabolite of glycerophospholipid, and trehalose, an intermediate of starch and sucrose metabolism, exhibited higher concentrations in aged worms compared with young worms. In addition, decreased concentrations of intermediates of purine metabolism were observed in aged worms, such as allantoin, glutamine, ADP and AMP. Other groups dysregulated and relating to aging included groups corresponding to aminoacyl-tRNA biosynthesis and glutamate metabolism, which include the varying levels of some amino acids (Table S2). In addition, variable concentrations of intermediates of pyrimidine metabolism and the citric acid cycle (TCA cycle) were observed, as illustrated in Figure 5. Overall, these metabolic pathway changes during aging suggested that aging is related to the progression of metabolism reprogramming.

### The long-lived *glp-1* mutants slowed the metabolic changes associated with aging

GLP-1 encodes a Notch family receptor that is essential for mitotic proliferation of germline cells. The *glp-1* loss-of-function (lf) mutants were long-lived when grown at the non-permissive temperature due to a failure of germline proliferation [34, 35]. To explore whether metabolites, which change in abundance with age in wild-type worms, display a slower rate of change when the lifespan is extended, we assessed the metabolic phenotype of *glp-1* mutants by combining NMR and UPLC-MS.

First, we analyzed the metabolome of 10A worms compared with YA worms in *glp-1* mutants. Our results revealed that the tendency of metabolite variation in *glp-1* mutants coincided with that detected in wild-type worms (Table S2), which indicates that the longevity mutants exhibit the same tendency of metabolic changes as the wild-type worms during aging.

**Figure 3:**
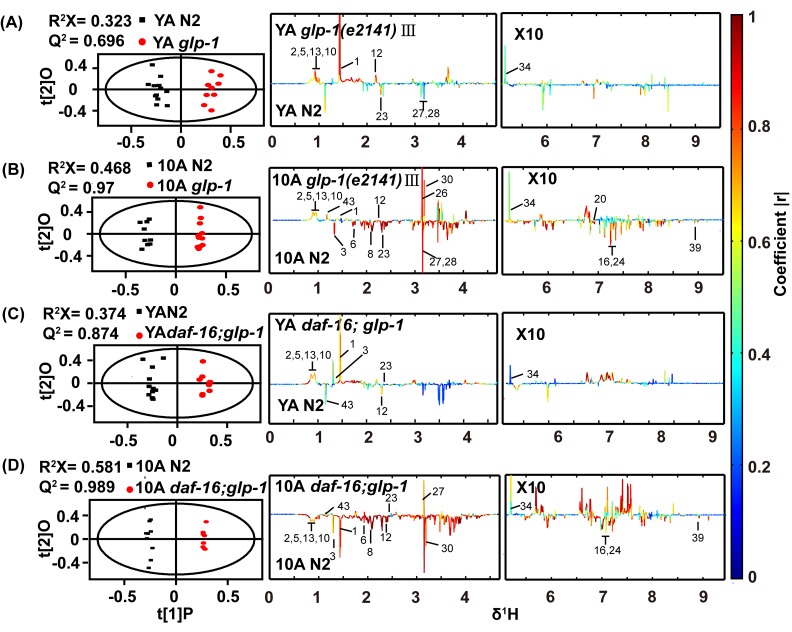
^1^H NMR-based metabolic profile analysis of the long-lived *glp-1(e2141)* mutants and the *daf-16(mu86);glp-1(e2141)* double mutants Scores and loading plots from OPLS-DA model of NMR data for (**A**) YA and (**B**) 10A wild-type N2 and *glp-1 (e2141),* (**C**) YA and (**D**) 10A wild-type N2 and *daf-16(mu86);glp-1(e2141)* double mutants. Detailed information about differences metabolites were summarized in the Table S3 and S4 (supplemental information).

Subsequently, we compared the metabolome of long-lived *glp-1* mutants and wild-type worms. The NMR data demonstrated that the metabolic profiles of the wild-type worms and g*lp-1* mutants were distinct, as revealed by the PCA score plot and the OPLS-DA analysis, for both YA and 10A worms (Figure 3A, 3B and Figure 6A), and the latter pattern displayed more obvious differences. These results may indicate that the metabolic differences are elevated between *glp-1* and wild-type worms as aging advances.

**Figure 4:**
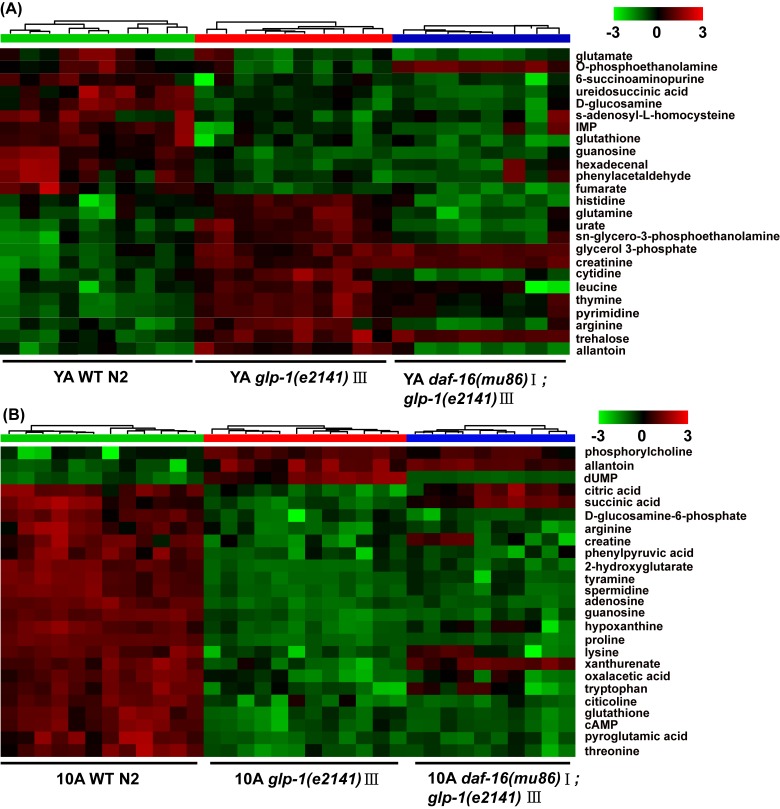
UPLC-MS-based metabolic profile analysis of the long-lived *glp-1(e2141)* mutants and the *daf-16(mu86);glp-1(e2141) double mutants* Metabolomics analysis from UPLC-MS data for (**A**) YA and (**B**) 10A wild-type N2 and *glp-1 (e2141)*, and for (**C**) YA and (**D**) 10A wild-type N2 and *daf-16(mu86);glp-1(e2141)* double mutants. Heatmap plot showed that 25 most importantly different metabolites from the comparison of the *glp-1* mutants and N2. More information was listed in the Table S3 and S4 (supplemental information). The detailed description of heatmap is as discussed in Figure 1B.

Distinct metabolic profiles of wild-type worms and g*lp-1* mutants were also obtained from the UPLC-MS data (data not shown). The top 25 significantly different metabolites from the UPLC-MS data of the YA or 10A *glp-1* mutants compared with wild-type worms are shown in Figure 4A and B. Our results displayed that some age-variant metabolites in the wild-type worms were dysregulated in the *glp-1* mutants (e.g., valine, GSSG, leucine, malate, serine), although some were not (e.g., cystathionine, glycine, arginine, trehalose). These results indicate that not all aspects of aging are reset in long-lived *glp-1* mutants. More detailed information regarding the significant metabolite changes in *glp-1* mutants compared to wild-type worms are listed in Tables S3 and S4.

**Figure 5:**
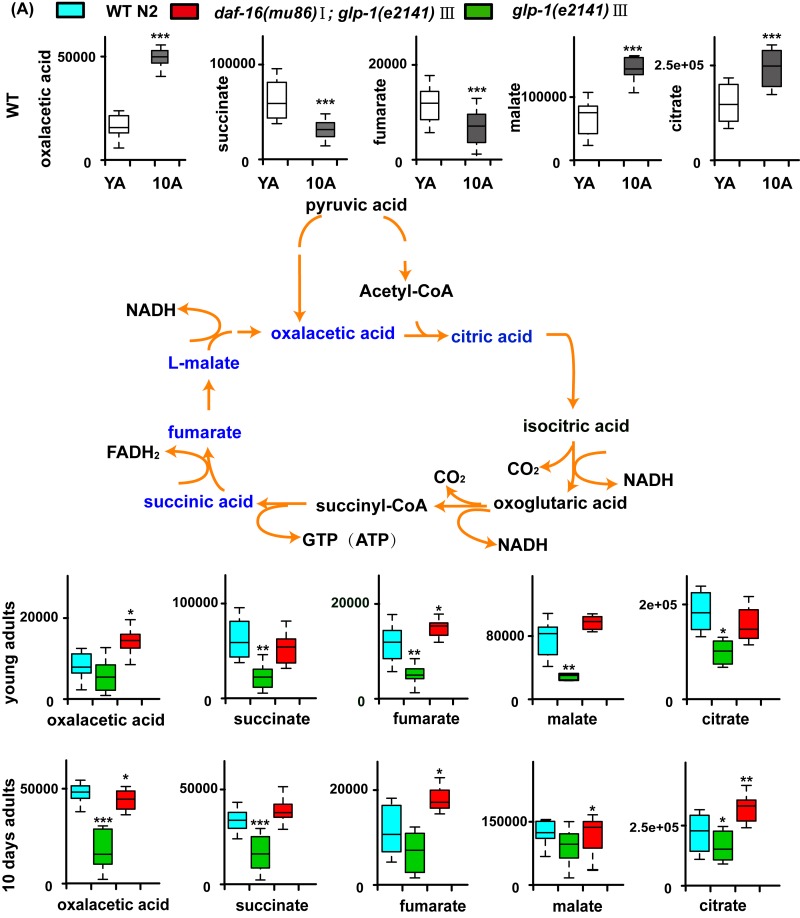

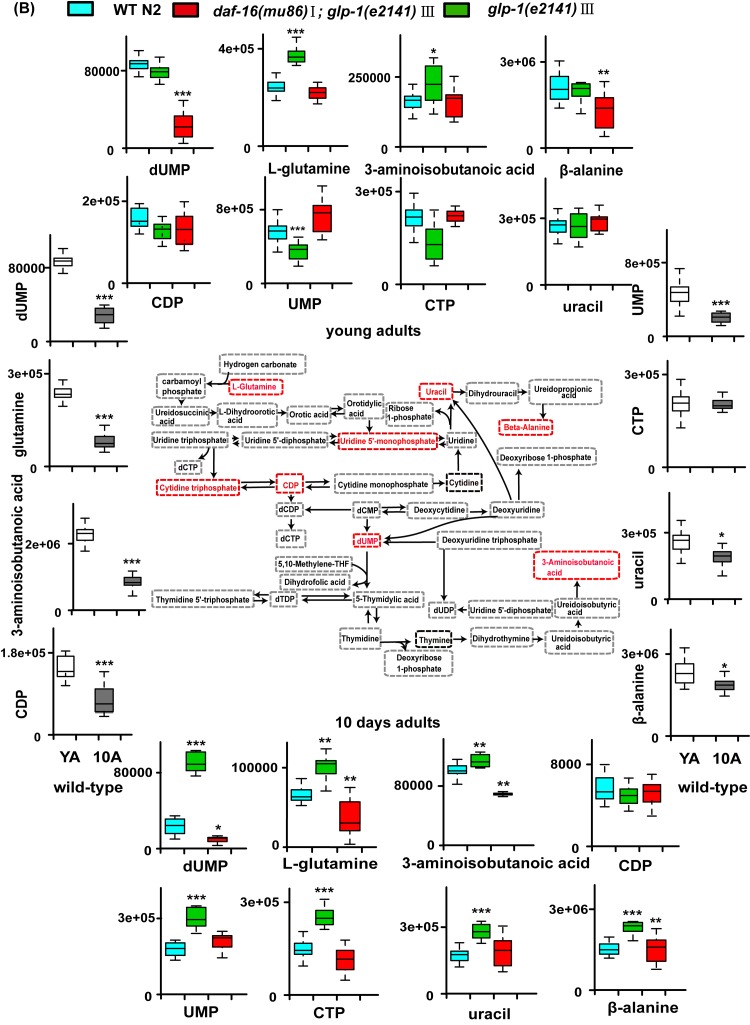
Models of aging-related changes in TCA cycle and pyrimidine metabolism (**A**) A summary of the biochemical pathway of TCA cycle metabolism alerted during aging, and *glp-1* against WT. In summary, with aging, accumulation of the TCA cycle intermediates such as citrate and malate suggests increased TCA cycle metabolism. Furthermore, in the long-lived *glp-1* mutants, the levels of TCA cycle intermediates decreased at stage of the young adults and 10-day adults compared with WT.

The metabolite set enrichment analysis and metabolome view analysis revealed that the metabolic pathways dysregulated by *glp-1* and related to age in wild-type worms were the TCA cycle, pyrimidine metabolism, glycerophospholipid metabolism, starch and sucrose metabolism and purine metabolism (Figure S2A and S2B). Our results confirmed that, for *glp-1* mutants compared with N2, the level of citric acid cycle intermediate metabolites (oxaloacetate, succinate, fumaric acid, malate and citrate) and glycerophosphi-lipid metabolism (e.g., phosphocholine) significantly decreased, and pyrimidine intermediate metabolites (e.g., dUMP, L-glutamine, 3-aminoisobutanoic acid, uracil, CDP and CTP), metabolites of starch and sucrose metabolism (e.g., trehalose) and metabolites of purine metabolism (e.g., allantoin), significantly increased (Figure 5A and B, Table S3 and S4). A previous analysis of the metabolome of wild-type N2 illustrated that, in aged worms, the levels of citric acid cycle and glycerophospholipid metabolism intermediates were higher, and those of pyrimidine and purine intermediates were lower (Figure 5 and Table S2). Taken together, these results indicate that long-lived *glp-1* mutants shift their metabolome toward a younger state.

### FOXO/DAF-16 mediated aging-related metabolic variations in *glp-1* mutants

**Figure 6:**
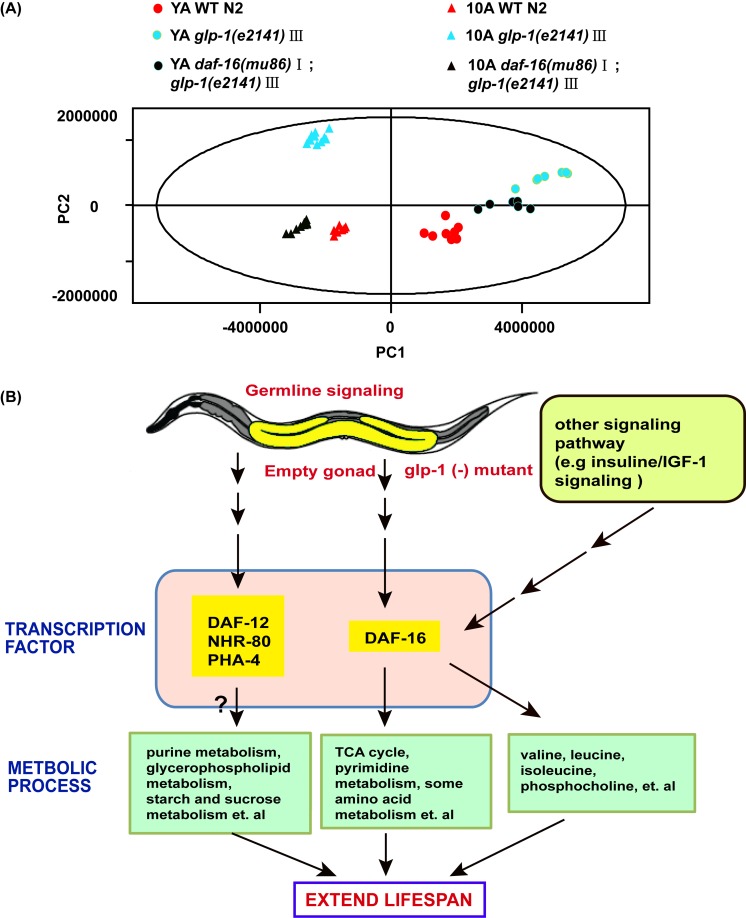
Aging and reproduction associated with metabolic variations in *C. elegans* (**A**) PCA included young adults and 10-day adults WT, *glp-1* and *daf-16;glp-1*. PC1 and PC2 stand for the first and second principal components, respectively. (**B**) Model on how germline less signals regulate metabolome of worms to attenuate aging. See detailed explanation in the main text of the discussion.

In characterizing the metabolic phenotype of *glp-1* related to anti-aging, we reasoned how germline signals regulate metabolism to extend lifespan. It was reminiscent of the Forkhead box O (FOXO) homolog DAF-16 transcription factor, which was shown to mediate the lifespan prolonging effect of germline-less *glp-1* mutants [34]. In addition, some evidence has indicated that FOXO/DAF-16 regulates the relationship between the longevity phenotype and maintaining lipid homeostasis in germline defect mutants [36]. To detect whether *glp-1* mutant modulation of the metabolism of other polar small molecules was dependent on DAF-16, we analyzed the metabolome of *daf-16;glp-1* double mutants. PCA analysis of the NMR data revealed a distinction between the *daf-16;glp-1* mutants and the N2 or *glp-1* YA or 10A worms (Figure 6A), and the metabolic pattern of 10A worms differed more than that of the YA worms. The supervised OPLS-DA model demonstrated that different metabolite levels existed between the double mutants and N2, and the results are shown in Figure 3C and D. Subsequently, the results of PCA and OPLS-DA analyses for the UPLC-MS data were in agreement with the NMR data and other results not shown.

Furthermore, the detailed analysis of different metabolites indicated that some dysregulated metabolites in the *glp-1* mutants were *daf-16* dependent (such as lysine, citric acid, leucine and dUMP) (Figure 4A and B, Tables S3 and S4), in which the metabolites upregulated in *glp-1* showed an insignificant difference or were moderately downregulation in the double mutants, and vice versa. In contrast, other metabolites displayed more complex epistatic patterns, such as trehalose, adenosine, IMP and guanosine (Figure 4A and B, Tables S3 and S4). This further illustrates that the *glp-1* mutants extend their lifespan by regulating downstream targets, including DAF-12, FOXA/pha-4 and NHR-49 besides DAF-16 [19, 36, 37], such that the regulation of metabolism in *glp-1* mutants is only partially dependent on DAF-16. Interestingly, after performing the metabolite set enrichment analysis and metabolome view analysis, we found that the TCA cycle and pyrimidine metabolism regulated by *glp-1* were mediated by DAF-16 (Figure 5A and B).

We also detected the metabolome of 10-day adult *daf-16*;*glp-1* mutants compared with young adult *daf-16*;*glp-1* mutants, and consistent results with wild-type were observed in the double mutants (Table S2, supplemental information).

## DISCUSSION

Researchers have recently dedicated considerable effort to the study of the mechanisms of longevity by utilizing OMIC approaches. In this work, we performed a metabolomics analysis by combining NMR and UPLC-MS. Our results indicated that the metabolome revealed promising metabolite markers involved in aging in *C. elegans*. Moreover, related metabolic pathway variations during aging were observed in glutathione metabolism, glutamate metabolism, taurine and hypotaurine metabolism, the TCA cycle, purine metabolism and pyrimidine metabolism and aminoacyl-tRNA biosynthesis. In addition, variations in trehalose and taurocholate were observed during aging. Furthermore, analyzing the metabotype of long-lived *glp-1* mutants suggested that germline-mediated longevity is inseparable from the regulation of age-related metabolic variations.

In *C. elegans*, previous studies have also analyzed metabolic changes during aging by NMR. For example, Clement and coworkers found that the concentrations of saturated and unsaturated lipids, glycerophosphocholine (GPC), phosphorycholine (PCho), glutamine, and glycine increased with age, while another 14 metabolites (alanine, arginine, isoleucine, leucine, lysine, phenylalanine, tyrosine, valine, formate, cystathionine, glutamate, acetate, lactate and glycerol) decreased [27]. Other researchers compared 10-day-old adult (10A) worms with young adult (YA) worms and found that the concentrations of acetate, arginine, asparagine, betaine, choline, cystathionine, glutamine, glycine, histidine, isoleucine, leucine, malate, phosphorycholine, putresine, serine, threonine, trehalose, valine and glycerophosphocholine were increased in the 10A worms, while those of alanine, glutamate, lactate, proline and succinate were decreased [38]. Furthermore, Neil Copes and co-works also uncovered metabolic changes during aging through an analysis metabolome of *glp-4* mutants cultured in liquid medium by GC-MS [39].

Most of these metabolomics studies with *C. elegans* have focused on finding biomarkers linked to senescence. However, the variation of metabolism during aging is a complex and sophisticated process. Recent work has suggested that, as individuals age, changes occur not only in the levels of specific molecules but also in the way that these molecules interact with one another within networks [40]. By contrast, our work discussed age-specific changes based on the network structure and illustrated that aging is characterized by metabolome remodeling. The metabolic pathways that we identified using our integrative approach also provide new insight into how the metabolic network is regulated by external and internal signals. In addition, our work was performed by combing NMR with UPLC-MS, which enhanced the coverage of the analysis.

In our analysis, we found that, compared with YA N2, the concentrations of glutathione (GSH), taurine and hypotaurine decreased, while oxidized glutathione (GSSG) increased in aging worms (Table S2). Glutathione and taurine are antioxidants, while oxidized glutathione is regarded as an indicator of increased oxidative stress. These results of decreased levels of GSH, taurine and hypotaurine and increased levels of GSSG with aging are consistent with previous studies in other model organisms [41–43], suggesting a vicious cycle of decreased antioxidant ability and increased oxidative stress that accompanies aging.

We also detected decreasing levels of the intermediates of purine metabolism (glutamine, ADP, AMP and allantoin) and pyrimidines intermediate metabolites (CDP, dUMP, glutamine, 3-aminoisobutanoic acid and CTP) in aged worms, which suggested the down-regulation of the purine and pyrimidine metabolism during aging (Figure 2 and Table S2). Previous studies have reported that many diseases such as Alzheimer's disease, immunodeficiency and growth retardation are related to disorders of purine and pyrimidine metabolism [44, 45]. Consistent with our results, the downregulation of purine and pyrimidine metabolism in aging mice was previously indicated in a transcriptional analysis [46].

We also observed that some citric acid cycle intermediate metabolites, such as oxaloacetate, succinate, fumarate, malate and citrate, were upregulated in aged animals (Figure 2, Figure 5A and Table S2). Other researchers found that several TCA cycle genes were upregulated in long-lived Ames dwarf mice and Little mice compared with wild-type controls [47]. In yeast, several TCA cycle gene knockouts exhibited markedly extended lifespans compared with wild-type under DR conditions [48]. In long-lived *C. elegans* dauer larvae, downregulation of the TCA cycle was observed [49]. A recent study showed that higher concentrations of TCA cycle intermediates were linked to shorten lifespans, possibly due to increased cardiovascular risk [23].

In addition, decreased cystathionine was observed in our aged worms (Figure 2B and Table S2). Polymorphisms in cystathionine beta synthase (CBS), which catalyzes the conversion of homocysteine to cystathionine, is well-known risk factor for Alzheimer's disease [50]. Our study also revealed that taurocholate, which is a bile acid conjugate of cholic acid and taurine, is a significant marker of longevity (Figure 2B and Table S2). Another recent study reported that increased taurocholate was related to reduce lifespan [23]. Taken together, these results demonstrate that aging is the result of the accumulation of molecular damage and reprogramming metabolism.

Furthermore, extending upon prior works, by analyzing the metabolome of *glp-1* mutants, we found that the longevity phenotype of *glp-1* mutants was inextricably linked to the metabotype. In *C. elegans*, the germline integrates nutrient signaling and communicates with other tissues to modulate aging. Previous studies have indicated that reproduction signals that affect aging were connected to the regulation of lipid metabolism, at least in part [51]. Another study reported the metabolic variations in *glp-1* mutants by using gas chromatography (GC) and LC-MS [52, 53].

We found that ermline-less mutants regulated some age-related metabolic pathways, including glycerophospho-lipid metabolism, the TCA cycle, starch and sucrose metabolism, purine metabolism and pyrimidine metabolism, to attenuate aging. It should be noted that, in our results, for pyrimidine metabolism, the level of some metabolites (e.g., such CDP, UMP, CTP) were not influenced by *glp-1* mutants in achieving the longevity phenotype at the young adult stage. This could be interpreted that these nucleotide metabolites are required for cell proliferation [54, 55], such that at the young adult stage, fertile N2 can upregulate the level of nucleotides to carry out DNA replication and egg-laying compared with infertile *glp-1*. Thus, our results did not show increased levels of these nucleotide metabolites in *glp-1* mutants at this stage. However, in day 10 of adult worms, the effect of egg-laying had been eliminated such that increased levels of CDP, UMP and CTP were observed in *glp-1* mutants compared with N2.

In addition, trehalose was also significantly accumulated in the *glp-1* mutants (Figure 4 and Table S3 and S4). Trehalose accumulation was identified as part of a longevity signature in long-lived mutants [25], and exogenous trehalose treatment could also extend lifespan in *C. elegans* [56]. Trehalose is a significant storage molecule, which is considered to be an important stress-responsive metabolite and longevity assurance sugar in *C. elegans* [49].

Among the age-related metabolic pathways regulated by *glp-1*, the TCA cycle and pyrimidine metabolism showed patterns of DAF-16 dependence (Figure 6B). The TCA cycle was downregulated in the *glp-1* mutants, but was not downregulated or moderately elevated in the *daf-16;glp-1* double mutants. A similar trend was found for pyrimidine metabolism. It should be noted that, theoretically, the metabolic pathways were elevated or repressed in the *glp-1* mutants but not in the *daf-16;glp-1* double mutants. However, in our study, the double mutants showed a slight trend of opposite regulation. This result could suggest that several upstream signals affect DAF-16 to regulate aging and that the signaling pathways and metabolic pathways are complex and engage in crosstalk. Furthermore, our results illustrate that the *glp-1* mutants extend lifespan by regulating various downstream targets, including DAF-12, FOXA/pha-4 and NHR-49, in addition to DAF-16 [19, 36, 37], such that some metabolic regulation in the *glp-1* mutants was independent of DAF-16, such as purine metabolism, starch and sucrose metabolism and glycerophospholipid metabolism (Figure 6B).

We also noted the limitations of this study. First, a previous study has reported that the use of 5′-fluorodeoxyuridine (FUdR) may have a significant effect on the levels of many metabolites (including betaine, lactate, NAD, glycerol, tyrosine, glutamate, glutamine and GPC) [57], but the biomass of worms required for metabolomic analysis cannot be collected without using FUdR or a similar intervention. In our study, in order to reduce the influence of FUdR, all strains were maintained on the same plates either with or without FUdR, and before collecting aged samples, the worms maintained on the plate containing FUdR were transferred to a plate without FUdR and maintained for two days.

Another limitation associated with the use of the *glp-1* strain is the required temperature shift of the eggs from 20°C to 25°C to induce sterility. This shift could affect stress resistance. Stress resistance is often directly proportional to longevity [58]. However, overall, in our trial, all *C. elegans* stains underwent the same treatment, which reduces the different effects of stress resistance on metabolism among the strains.

Comprehensive metabolome analyses often observe pairs of metabolites that are correlated across samples but which are apparently unrelated to the metabolic reaction. In the future, a study combining untargeted metabolomics with targeted metabolomics is necessary to exclude the unrelated information. More challenging, it remains to be seen whether age-related changes in metabolite correlations can be altered through genetic or pharmacological approaches or by consuming meta-bolites whose correlations change with age to further verify the discovery linked to aging by metabolomics.

Overall, aging is a complicated process related to cellular metabolism, and systems approaches such as comprehensive non-targeted metabolomics have the potential to disclose the linkage between metabolic changes with aging and varying stress resistance. This study combined NMR and UPLC-MS technology to display a comprehensive and unbiased footprint of the metabolic variations that accompany aging in different mutants at the whole organism level in *C. elegans*. Comparing young adults with older adults among different strains, we found that age-varying metabolic pathways included glutathione metabolism, glutamate metabolism, purine metabolism and pyrimidine metabolism, taurine and hypotaurine metabolism, and the TCA cycle, suggesting that aging emerges as a metabolome remodeling process and the accumulation of damage. In addition, in long-lived *glp-1* mutants, we observed different metabolic characteristics compared with N2, and the metabolic differences were more marked in old stage worms than in YA worms, indicating that aging is associated with increasing metabolite diversity. Moreover, our results showed that long-lived *glp-1* mutants influenced some aging-related metabolic variation, which demonstrates that the longevity phenotype of the *glp-1* mutants is associated with its metabotype. Additionally, we found that the TCA cycle and pyrimidine metabolism was particularly significantly associated with the longevity regulated by germline-less signals, which is mediated by downstream DAF-16 transcription factors. Altogether, these results demonstrate that high-sensitivity metabolomic studies have excellent potential not only to reveal mechanisms leading to senescence but also to help reveal pathways associated with overall survival and longevity. Therefore, in subsequent studies, extending the analysis to lipidomics and combining other molecular level analyses, including transcriptomic or proteomic analyses, will provide further insight into the interrelationships between metabolic function with respect to longevity, as well as the association between germline-less signal regulation longevity and metabolism.

## MATERIALS AND METHODS

### Culture of nematodes

Wild-type N2, CF1903 *glp-1(e2141)III*, and CF1880 *daf-16(mu86)I;glp-1(e2141)III* were from the Caenorhabditis Genetics Center (CGC), and maintained under standard conditions on nematode growth media (NGM) with *Escherichia coli* OP50 as described previously, unless otherwise stated [59].

### Sample preparation for metabolomic analysis

To reduce variation related to sample preparation and analysis, trials were performed on a large sample of worms (~8000) and prepared in at least two independent experiments. All animals were synchronized, and the eggs were then incubated at 20°C overnight in M9 buffer and grown on NGM plates. For *glp-1(e2142)* and *daf-16(mu86);glp-1(e2141)* alleles corresponding to temperature-sensitive mutants, synchronized L1 larvae were incubated at 20°C for 12 h, then transferred to 25°C to eliminate germ cells, and finally returned to 20°C at the young adult stage. Other worms were treated via the same procedures. For collection of old worms, worms reaching the late L4 stage or the young adult stage were moved to plates containing 10 μM 5-fluoro-2′-deoxyuridine (FUdR, Sigma) to prevent self-fertilization. This protocol allowed for the maintenance of synchronized populations until old age. To ensure enough food and fresh media, the worms were transferred to fresh plates every two days by washing with M9 buffer. In addition, the worms at day 7 of adulthood (at the end of the egg-laying period) were transferred from plates containing 10 μM FUdR to plates without FUdR until day 10 for harvesting. Collection of young adult (YA) or day 10 adult (10A) worms was performed on the same day for all genotypes. For each biological replicate, ~8000 animals were pooled and washed with M9 buffer. All samples were snap frozen in liquid nitrogen and dried overnight in *vacuo* at a low temperature, weighted and stored at -80°C until extraction.

Following the method describe by Geier et al [60], metabolites from *C. elegans* samples were extracted three times with 600 μL of precooled MeOH/H_2_O (4:1) using a TissueLyser at 55 Hz for 90 s. All extracts were subjected to centrifugation (12000 rpm for 10 min at 4°C). The collected supernatants were split into two aliquots at a ratio of 1:6 for UPLC-MS and NMR analyses. The samples for UPLC-MS were further split into two equal parts for analysis in positive and negative ionization mode. All samples were stored at -80°C until analysis.

### Analytical procedures for ^1^H NMR analysis

#### Sample preparation

For NMR, the supernatant was evaporated to dryness with a vacuo at a low temperature and dissolved in 550 μL phosphate buffer (0.1 M, K_2_HPO_4_/NaH_2_PO_4_, pH 7.4) containing 99.9% D_2_O and 0.01% NaN_3_ and then transferred to a 5-mm NMR tube for NMR analysis.

#### Apparatus and analytical conditions

All samples were analyzed using an 800 MHz Bruker Avance spectrometer (800.3 MHz for proton frequency) equipped with a QCI-P cryoprobe at 298 K. For each sample, a standard 1D ^1^H NMR spectrum was acquired by using a water suppression pulse sequence with water irradiation during the relaxation delay and mixing time (the standard ‘noesypr1d’ in the Bruker library). For all experiments, 64 transients were collected with 64 K data points and a spectral width of 20 ppm. For metabolite assignment, a set of two-dimensional (2D) NMR spectra was acquired, including ^1^H J-resolved, ^1^H-^1^H COSY and TOCSY, ^1^H-^13^C HSQC and HMBC spectra and analyzed according to the procedure reported previously [61].

#### Data processing

All spectra were baseline- and phase-corrected manually through TOPSPIN (V3.2, Bruker Biospin, Germany) and then referenced to the signal of trehalose (δ5.19). All the spectra were then integrated into regions by dividing into bins with a bucket-width of 0.004 ppm using the AMIX package (V3.9.14, Brucker Biospin, Germany) and then normalized against the dry weight of the samples. Metabolite assignment was conducted by combining two-dimensional (2D) NMR spectral with reference data from the literature [25, 38], the HMDB [62], and MMCD [63].

### Analytical procedures for LC-MS analysis

#### Sample preparation

Samples were thawed at room temperature, centrifuged at 12,000 rpm for 10 min at 4°C, and analyzed by UPLC-MS.

#### Apparatus and analytical conditions

Liquid chromatography was performed using a reversed-phase C18 column (ACQUITY UPLC HSS T3, waters, 1.8 μm, 150×2.1 mm diameter column) with a flow rate of 300 μL/min at 35°C, and 8 μL of sample was injected. Eluent A was water and eluent B was ACN, both with 0.1% formic acid. The initial eluent consisted of 2% solvent B; the percent of buffer B was then gradually increased to 100% in 20 min, held there for 5 min, and then returned to the initial condition in 0.1 min. The column was re-equilibrated for 4.9 min, and the total run time was 30 min. Analyses were conducted using an Agilent 1290 UPLC (Agilent, Santa Clara, CA) system connected to an Agilent 6500 Q-TOF Mass Spectrometer (Agilent, Santa Clara, CA).

Mass spectrometry analyses were conducted in either positive or negative ion mode with a cone voltage of 3500 V. The drying gas temperature was 350°C with a flow of 9 L/min, and the nebulizer was set to 35 psig. Spectra were collected at a mass range of 80-1500 m/z. The mass analyzer had a mass accuracy of approximately 2 ppm after the calibration tests.

#### Data processing

Data files from the UPLC-MS were converted to mzData format using the Masshunter Qualitative software provided with Agilent instruments (Agilent Sana Clara, CA). The data were analyzed by using the open-source software XCMS and CAMERA implemented with the freely available R statistical language (version 3.2.2). The procedures and parameters used for the alignment of features were followed according to previous studies, with some modifications [64]. Identification of metabolites was performed based on their molecular ion masses and MSn fragmentation compared with the literature and metabolomic library entries of purified standards. Subsequently, the putative identifications were verified by comparing with the retention time matches to those of authentic standard compounds. In addition, we also purchased some commercially available purified standard compounds to identify metabolites when necessary.

### Bioinformatics and statistics

For NMR data, multivariate data analyses were performed by SIMCA-P11.5 (Umetrics, Sweden), and the analytical method was modified from previously published studies [65]. Briefly, after scaling the data to unit variance, PCA and OPLS-DA were conducted. The loading plots from these models were generated by using an in-house developed MATLAB script after back-transformation, where the signals were color-coding with correlation coefficients to reveal significantly altered metabolites.

For the UPLC-MS data, after normalizing against the dry weights, the resolved data sets were subjected to statistical analysis based on the algorithm of significance analysis of microarray (SAM) data (the false discovery rate FDR ≤0.05 unless otherwise noted) by using the web-based metabolomics data processing tool metaboAnalyst [66] and OPLS-DA by SIMCA-P11.5. The potential differential metabolites provided by OPLS-DA or SAM were further analyzed by Mann-Whitney U tests using PASW Statistics 20 (SPSS, Chicago, USA), and a p-value of 0.05 or less was considered significant. Finally, the significantly different metabolites were subjected to metabolite set enrichment analysis (MSEA) and pathway analysis using metaboAnalyst as described in previous studies [22]. Unsupervised hierarchical clustering was performed by using complete linkage and the Pearson rank correlation distance, and a heat map of the different metabolites was plotted using metaboAnalyst.

## SUPPLEMENTARY INFORMATION


