# Supplementary material for: Metabolomic signature associated with reproduction-regulated aging in Caenorhabditis elegans
Source: Aging (Albany NY). 2017 Feb 6;9(2):447–63. doi: 10.18632/aging.101170 (PMC5361674; doi:10.18632/aging.101170)
Supplement: Supplementary file 1 [file aging-09-447-s001.pdf]

SUPPLEMENTARY INFORMATION

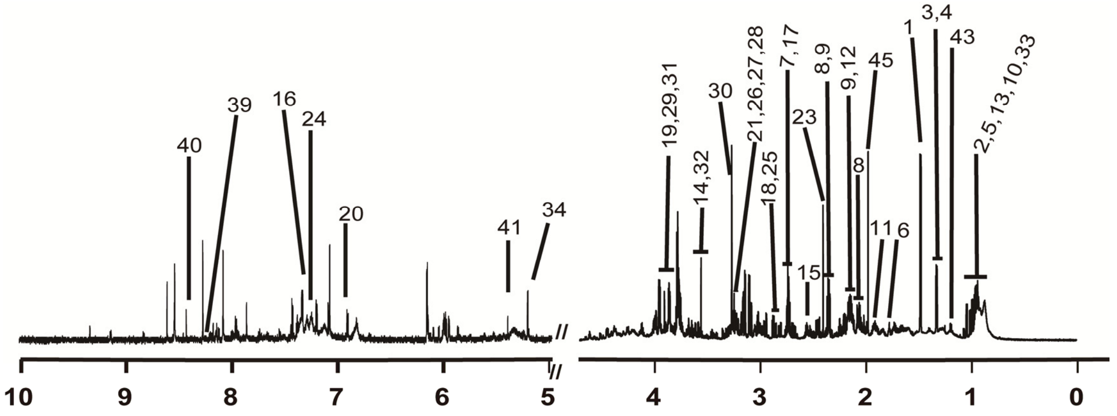

Figure S1. Typical 800MHz ^{1}H NMR spectra of *C. elegans*. Metabolites keys were showed in the Table S1 (supplemental information).

Table S1. ^{1}H and ^{13}C NMR data for metabolites keys: GPC, glycerophosphocholine; GSSG, glutathione disulfide; #, not determined.

keys	metabolites	moieties	δ1H (multiplicity)	δ 13C
**1**	alanine	αH	3.77(q)	53.4
		βH	1.48(d)	19.1
**2**	isoleucine	αCH	3.65(d)	62.4
		βCH	1.97(m)	38.7
		γCH_2	1.47(m)	27.4
		$\gamma' CH_3$	1.007(d)	17.6
		δCH_3	0.94(t)	13.9
**3**	threonine	αCH	3.58(d)	63.3
		βCH	4.25(m)	68.8
		γCH_3	1.33(d)	22.3
**4**	lactic acid	αCH	4.11(q)	71.4
		βCH	1.33(d)	23
**5**	valine	αCH	3.61(d)	63.2
		βCH	2.26(m)	31.9
		γCH_2	1.04(d)	20.8
		$\gamma' CH_3$	0.99(d)	19.5
**6**	lysine	αCH	3.75(t)	57.5
		βCH_2	1.82(m)	32.8
		γCH_2	1.47(m)	24.3
		δCH_2	1.72(m)	29.3
		εCH_2	3.02(t)	41.9
**7**	methionine	αCH	3.86(t)	56.6
		βCH_2	2.17(m)	33
		γCH_2	2.64(t)	31.7
		εCH_3	2.12(s)	16.8
**8**	proline	αCH	4.14(dd)	64
		βCH_2	2.06(m)	31.9
		βCH_2	2.35(m)	31.9
		γCH_2	2.01(m)	26.6
		δCH_2	3.42(m)	48.9
		δCH_2	3.34(m)	48.9

keys	metabolites	moieties	δ1H (multiplicity)	δ 13C
**9**	glutamate	αCH	3.75(m)	57.5
		βCH_2	2.07(m)	29.8
		βCH_2	2.15(m)	29.8
		γCH_2	2.35(m)	36.3
**10**	α-aminobutyric acid	αCH	3.71(t)	58.7
		βCH_2	1.91(m)	26.6
		γCH_2	0.97(t)	11.4
**11**	acetic acid	CH3	1.91(s)	26.2
**12**	glutamine	αCH	3.77(t)	56.9
		βCH_2	2.14(m)	29.2
		γCH_2	2.45(m)	33.7
**13**	leucine	αCH	3.76(t)	56.3
		βCH_2	1.71(m)	42.6
		γCH	1.72(m)	26.7
		δCH_3	0.96(q)	23.8
		δ'CH3	0.95(q)	24.9
**14**	glycine	αCH	3.55(S)	44.3
**15**	β-alanine	αCH2	2.55(t)	36.42
		βCH_2	3.17(t)	39.4
**16**	phenylalanine	αCH	3.99(dd)	58.9
		βCH_2	3.14(q)	39.3
		βCH_2	3.28(q)	39.3
		δ&δ'CH	7.38(m)	130.5
		ε&ε'CH	7.41(m)	131.9
		ζCH	7.31(d)	129.7
**17**	aspartic acid	αCH	3.89(dd)	55
		βCH_2	2.68(dd)	39.4
		βCH_2	2.81(dd)	39.4
**18**	asparagine	αCH	3.98(dd)	54.1
		βCH_2	2.86(dd)	37.4
		βCH_2	2.95(dd)	37.5
**19**	serine	αCH	3.84(dd)	59.2
		βCH_2	3.95(m)	63.1
**20**	tyrosine	δCH,δ'CH	6.91(d)	118.9
		εCH,ε'CH	7.20(d)	133.7
**21**	arginine	αCH	3.77(t)	56.9
		βCH_2	1.91(m)	30.4
		γCH_2	1.65(m)	26.7
		δCH_2	3.24(t)	43.3
**22**	ethanolamine	1CH2	3.82(t)	60.5
		2CH2	3.14(t)	44.1
**23**	succinate	CH2	2.4(s)	37.1
**24**	tryptophan	αCH	4.06(dd)	57.9
		βCH_2	3.48(dd)	29.3
		βCH_2	3.31(dd)	29.3
		δCH	7.33(s)	127.9
		ε'CH	7.26(m)	124.7
		ζCH	7.54(d)	114.8
		ζ'CH	7.17(m)	122
		ηCH	7.74(d)	121.1
**25**	cystathionine	2CH	3.85(dt)	56.6
		3CH2	2.15(m)	32.6
		4CH2	2.73(m)	30
		6CH2	3.11(m)	34.9
		7CH2	3.95(dt)	56.4
**26**	choline	1CH2	3.52(dd)	70.3
		2CH2	4.06(m)	57.9
		CH3	3.2(s)	56.7

keys	metabolites	moieties	δ1H (multiplicity)	δ 13C
**27**	phosphorylcholine	1CH2	3.6(m)	69.3
		2CH2	4.16(m)	60.9
		CH3	3.21(s)	56.7
**28**	GPC	1,2CH2	3.91(m)	#
		1',3CH2	3.68(m)	#
		2'CH2	4.34(m)	68.9
		CH3	3.21(s)	56.9
**29**	3-phosphoglyceric acid	2CH	4.2(dd)	75.8
		3CH2	3.87(m)	69.7
		3CH2	4.03(m)	69.7
**30**	betaine	CH2	3.9(s)	69
		CH3	3.26(s)	56.2
**31**	glycerol-3-phosphate	1CH2	3.61(dd)	64.8
		1CH2	3.67(dd)	64.8
		2CH	3.82(m)	74.2
		3CH2	3.82(m)	67.7
**32**	glycerol	1,3CH2	3.65(m)	65.4
		1,3 CH2	3.77(m)	65.4
		CH	3.55(m)	75
**33**	3-hydroxyisobutyric acid	2CH	2.47(m)	47.7
		3CH2	3.52(m)	67.6
		3CH2	3.70(m)	67.6
		CH3	1.06(d)	16.9
**34**	trehalose	1,1'CH	5.19(d)	96.078
		2,2'CH	3.64(dd)	73.884
		4,4'CH	3.45(t)	72.537
**35**	fumarate	CH	6.51(s)	138
**36**	O-phosphoethanolamine	1CH2	3.96(m)	63.1
		2CH2	3.21(t)	43.5
**37**	uracil	5CH	5.81(d)	103.8
		6CH	7.52(d)	146.3
**38**	GSSG	2CH	3.77(m)	56.9
		$3CH_2$	2.14(q)	29.2
		$4CH_2$	2.54(m)	34.2
		$7CH_2$	4.77(dd)	55.5
		$10CH_2$	3.77(m)	46.3
		$12CH_2$	3.3(dd)	41.6
		$12CH_2$	2.97(dd)	41.6
**39**	niacinamide	2CH	8.92(s)	150.4
		4CH	8.24(dd)	139.2
		5CH	7.58(dd)	126.8
		6CH	8.70(dd)	154.5
**40**	formic acid	CH	8.45(s)	172.4
**41**	allantoin	CH	5.39(s)	66.2
**42**	malate	2CH	4.29(dd)	73.4
		3CH2	2.66(dd)	45.6
		3CH2	2.35(dd)	45.6
**43**	3-aminoisobutyric acid	2CH	2.6(m)	42.3
		3CH2	3.05(dd)	45.2
		3HC2	3.09(dd)	45.2
		CH3	1.19(d)	18
**44**	pyroglutamic acid	2CH	4.17(dd)	60.9
		3CH2	2.51(m)	28.2
		3CH2	2.02(m)	28.2
		4CH2	2.4(m)	32.6
**45**	N-acetyl glutamic acid	CH3	1.98(s)	

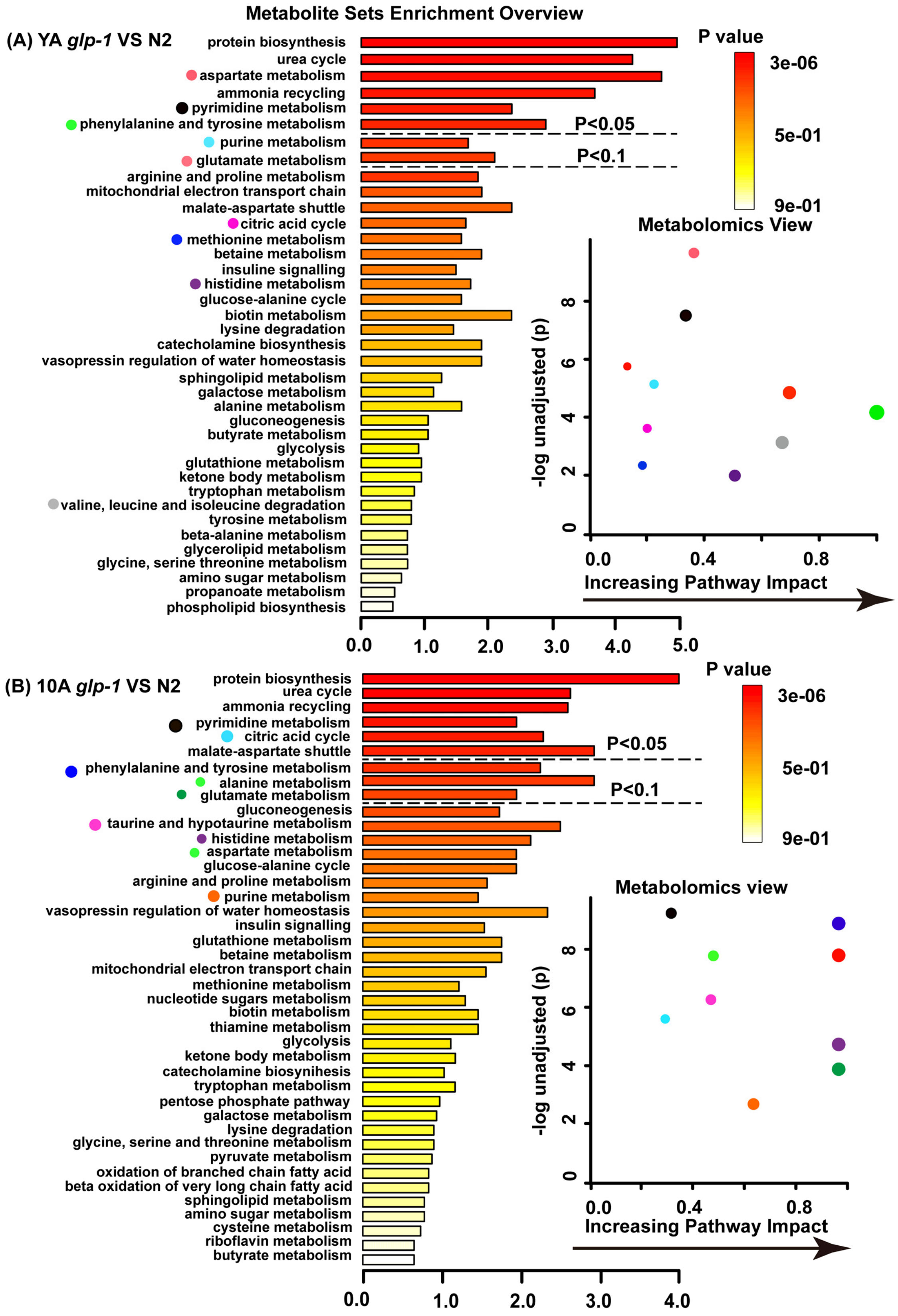

Figure S2. Pathway analysis for *glp-1* against wild-type. Summary plot for metabolite enrichment analysis(MSEA) (left panel) and metabolome view as discussed in Figure 2A, which showed the different metabolites when *glp-1* compared with N2 in young adult **(A)** and days 10 of adulthood **(B)**. Detailed description showed in the Figure 2A.

Table S2. Summary metabolite variations with age in WT, *glp-1(e2141)*, and *daf-16(mu86);glp-1(e2141)* double mutants.

metabolites	N2 10A VS YA		*glp-1* 10A VS YA		*daf-16;glp-1* 10A VS YA	
	P **value**	**Change**	***P*** **value**	**Change**	***P*** **value**	**Change**
cystathionine * LC	<0.0001	-0.8536	<0.0001	-0.9591	<0.0001	-0.9772
cytidine * LC	<0.0001	-0.5624	0.0101	-0.5987	0.0301	-0.3184
dUMP * LC	0.0549	-0.2056	<0.0001	-0.5007	0.0002	-0.5052
oxidized glutathione * LC	0.0085	0.7389	0.0390	0.4531	0.0321	0.3816
glycine* LC & NMR	0.0010	-0.8517	0.0002	-0.9266	<0.0001	-0.8778
hypotaurine * LC	<0.0001	-0.4369	<0.0001	-0.6298	<0.0001	-0.6381
glutamate* LC & NMR	<0.0001	-0.7660	<0.0001	-0.8488	<0.0001	-0.8427
glutamine* LC & NMR	<0.0001	-0.5738	<0.0001	-0.8334	0.0007	-0.8028
malate* LC & NMR	0.0002	0.6807	0.3496	0.0530	0.0435	0.3418
serine* LC & NMR	<0.0001	-0.6832	<0.0001	-0.8048	0.0005	-0.8294
taurine * LC	<0.0001	-0.8990	<0.0001	-0.8495	0.1393	-0.3810
taurocholate * LC	<0.0001	2.0653	<0.0001	2.4940	0.0157	0.4020
thymine * LC	<0.0001	-0.7643	<0.0001	-0.8712	<0.0001	-0.9298
citrate* LC & NMR	0.0002	0.4102	0.0001	0.3591	<0.0001	0.5374
fumarate* LC & NMR	0.0002	-0.3579	0.5667	0.0351	0.0815	-0.1645
glutathione* LC & NMR	0.0025	-0.2632	<0.0001	-0.5580	0.0781	-0.3395
arginine* LC & NMR	<0.0001	0.6144	0.0001	0.1869	0.0967	0.0654
histidine* LC & NMR	<0.0001	0.7263	<0.0001	0.2787	<0.0001	-0.3609
threonine* LC & NMR	<0.0001	0.7598	<0.0001	0.3656	<0.0001	-0.2541
aspartate* LC & NMR	0.1261	0.1738	<0.0001	0.6463	0.0011	-0.1360
oxaloacetate * LC	0.0185	0.1871	<0.0001	0.9707	0.0100	0.2146
leucine* LC & NMR	<0.0001	-0.4603	<0.0001	-0.4824	<0.0001	-0.6050
isoleucine* LC & NMR	0.0002	-0.3076	<0.0001	-0.3752	<0.0001	-0.4152
valine* LC & NMR	0.0213	-0.1947	0.0002	-0.3366	0.0003	-0.3629
3-aminoisobutyric acid* LC & NMR	<0.0001	-0.6261	<0.0001	-0.4931	<0.0001	-0.7728
alanine* LC & NMR	0.0008	-0.3545	<0.0001	-0.5765	<0.0001	-0.6421
asparagine* LC & NMR	0.1261	0.1738	<0.0001	0.6463	0.0011	-0.1360
succinate* LC & NMR	<0.0001	-0.6252	<0.0001	-0.7173	<0.0001	-0.6711
lysine* LC & NMR	0.1987	0.1468	0.0001	-0.4218	<0.0001	-0.3101
phosphorylcholine* LC & NMR	0.8637	0.0835	0.0006	3.0821	0.0991	0.7429
trehalose* LC & NMR	<0.0001	1.7455	<0.0001	1.1126	<0.0001	0.8642
Phenylalanine* LC & NMR	0.2481	-0.1056	0.0074	-0.2568	0.3301	-0.0689
isocitric acid * LC	0.0002	0.7665	<0.0001	1.2223	<0.0001	0.8032
ADP * LC	0.0008	-0.44527	0.003689	-0.4256	0.0034	-0.4413
AMP * LC	0.0003	-0.79027	<0.0001	-0.7351	0.0205	-0.7389
allantoin* LC & NMR	0.0076	-0.522	0.0464	-0.207	0.0003	-0.652
CDP * LC	<0.0001	-0.574	<0.0001	-0.5013	0.0125	-0.4269

metabolites	N2 10A VS YA		*glp-1* 10A VS YA		*daf-16;glp-1* 10A VS YA	
	***P* value**	**Change**	***P* value**	**Change**	***P* value**	**Change**
UMP * LC	<0.0001	-0.629	0.3976	-0.1236	0.0114	-0.557
CTP * LC	0.741	0.053	0.2067	0.002	0.0274	-0.255
uracil * LC	0.037	-0.29078	0.8383	0.0297	<0.0001	-0.53545
β-alanine* LC & NMR	0.0217	-0.2163	0.5291	0.0453	0.5086	-0.0774

Asteriks (*) denotes metabolites are verified by reference standards. Superscript letter LC indicated metabolites were detected with UPLC-MS platforms. *P* values were calculated by Mann-Whitney U test, and the *p*-value of 0.05 or less was considered significant. All statistical were calculated by using SPSS package.

Table S3. List of altered metabolites of each mutant strains against the wild type in young adults.

metabolites (young adult)	*P* value *glp-1* VS N2	*P* value *daf-16;glp-1* VS N2
2-oxoglutarate * LC	0.014	0.003
2-oxosuccinamate * LC	0.041	0.184
3-aminoisobutanoic acid * LC & NMR	0.0191	0.0904
3-hydroxydodecanoic acid * LC	0.231	0.732
3-indolepropionic acid * LC	0.468	0.003
3-sulfinoalanine * LC	0.035	<0.0001
5-hydroxy-L-tryptophan	<0.0001	<0.0001
6-succinoaminopurine * LC	0.001	<0.0001
9-hexadecenoylcarnitine * LC	0.03	0.048
adenine * LC	0.021	0.792
adenosine * LC	0.024	<0.0001
ADP * LC	0.048	0.001
alanine * LC & NMR	<0.0001	<0.0001
allantoin * LC & NMR	<0.0001	<0.0001
alpha-aminobutyric acid * NMR	0.025	0.235
alpha-lactose * LC	<0.0001	0.166
AMP * LC	0.007	0.391
androsterone * LC	0.553	0.025
arginine * LC & NMR	0.006	0.007
argininosuccinic acid * LC	0.048	<0.0001
ascorbate * LC	0.323	0.002
asparagine * LC & NMR	0.429	0.001
aspartate * LC & NMR	<0.0001	<0.0001
atenolol * LC	0.429	0.692
betaine * LC & NMR	0.119	0.931
biotin * LC	0.029	0.063
cAMP * LC	0.439	<0.0001
carnitine * LC	0.025	0.021
CDP-choline/Citicoline * LC	0.048	0.099
choline * LC & NMR	0.03	0.262

metabolites (young adult)	*P* value *glp-1* VS N2	*P* value *daf-16;glp-1* VS N2
citrulline * LC	0.007	0.003
creatinine * LC	<0.0001	<0.0001
cystathionine * LC & NMR	0.573	0.239
cysteate * LC	0.041	0.391
cysteine * LC	0.742	<0.0001
cytidine * LC	<0.0001	<0.0001
cytosine * LC	<0.0001	0.147
deoxyadenosine * LC	0.086	0.644
deoxycorticosterone * LC	0.692	0.012
D-fructose 6-phosphate * LC	0.51	0.598
D-glucosamine * LC	0.006	0.005
D-gluconic acid * LC	0.159	0.342
D-glucose * LC	<0.0001	0.009
dUMP * LC	0.573	<0.0001
D-xylonate * LC	0.114	0.008
ethanolamine phosphate * LC	0.356	0.005
FMN * LC	0.439	0.011
fumarate * LC & NMR	0.011	0.044
gamma glutamyl ornithine * LC	0.51	0.048
gamma-aminobutyric acid * LC	0.166	0.51
glucosamine * LC	0.015	0.007
glucose 6-phosphate * LC	0.049	0.002
glutamine * LC & NMR	0.002	0.0968
glutamate * LC & NMR	0.005	0.001
glutathione* LC	0.0105	<0.0001
glyceric acid * LC	0.235	0.012
glycerol * LC & NMR	0.005	0.018
glycerol-3-phosphate * LC & NMR	<0.0001	<0.0001
glycerophosphocholine * LC & NMR	0.345	0.196
guanosine * LC	<0.0001	0.001
hexadecenal * LC	<0.0001	0.429
histidine * LC & NMR	0.002	0.018
homocarnosine * LC	0.356	<0.0001
hydroxypyruvate * LC	0.778	0.007
hypotaurine * LC	0.048	0.01
hypoxanthine * LC	0.398	0.007
IMP * LC	0.001	0.391
inosine * LC	0.526	0.849
isocitric acid * LC	0.262	0.001
isoleucine * LC & NMR	0.0099	0.0166
kynurenine * LC	0.099	0.021
leucine * LC & NMR	<0.0001	0.742
leukotriene E4 * LC	0.888	0.006
lysine * LC & NMR	0.099	0.075
malate * LC & NMR	0.007	0.077
mannitol * LC	0.018	0.692

metabolites (young adult)	*P* value *glp-1* VS N2	*P* value *daf-16;glp-1* VS N2
mannitol-1-phosphate * LC	0.356	0.323
mannobiose * LC	<0.0001	0.468
methionine * LC & NMR	0.391	0.001
N-acetyl-L-glutamate* LC & NMR	<0.0001	0.001
N-acetylmethionine * LC	<0.0001	0.003
N-acetylputrescine * LC	0.002	<0.0001
NAD+ * LC	0.21	0.025
O-butanoylcarnitine * LC	**0.002**	**0.391**
O-phosphoethanolamine * LC	0.002	0.018
orotate * LC	0.778	0.03
oxaloacetate * LC	0.105	0.017
palmitoylcarnitine * LC	0.025	0.129
phenylacetaldehyde * LC	0.018	0.947
phenylalanine * LC & NMR	0.041	0.425
phosphorylcholine * LC & NMR	0.004	0.008
proline * LC & NMR	0.391	0.012
purine * LC	0.51	0.048
pyrimidine * LC	<0.0001	0.021
s-adenosyl-L-homocysteine * LC	0.01	0.742
serine * LC & NMR	0.006	0.044
sn-glycero-3-phosphoethanolamine * LC	0.003	0.356
succinate * LC & NMR	0.0003	0.518
taurine * LC	0.778	0.97
taurocholate * LC	0.187	0.025
threonine * LC & NMR	0.139	0.02
thymine * LC	<0.0001	0.0685
trehalose * LC & NMR	<0.0001	0.025
tryptophan * LC & NMR	0.468	0.003
tyrosine * LC & NMR	0.035	0.323
urate * LC	<0.0001	0.025
ureidosuccinic acid * LC	0.018	0.004
uridine * LC	0.008	0.01
urocanic acid * LC	0.742	0.035
valine * LC & NMR	0.03	0.895
xanthurenic acid * LC	0.006	0.553

Asteriks (*) denotes metabolites are verified by reference standards. Superscript letter LC indicated metabolites were detected with UPLC-MS platforms. Metabolite abundance level were reflected using colors, and with yellow being lower and red higher when mutants VS. N2. *P* values were calculated by Mann-Whitney U test, and the *p*-value of 0.05 or less was considered significant. All statistical were calculated by using SPSS package.

Table S4. List of altered metabolites of each mutant strains against the wild type in days 10 of adult worms. Detailed description showed in the Table S3.

metabolites (10-day-adult)	*P* value<br>*glp-1* VS N2	*P* value<br>*daf-16;glp-1* VS N2
2-hydroxyglutarate	<0.0001	<0.0001
3-aminoisobutanoic acid * LC & NMR	0.0049	0.0007
3-hydroxydodecanoic acid * LC	0.0008	0.0003
3-indolepropionic acid * LC	0.1842	0.0069
3-sulfinoalanine * LC	0.7728	<0.0001
4-aminobutanoate * LC	<0.0001	<0.0001
5-hydroxy-L-tryptophan * LC	0.0001	<0.0001
6-succinoaminopurine * LC	0.0022	0.0001
acetamidopropanal * LC	<0.0001	<0.0001
acetylcarnitine * LC	0.0002	0.3913
adenine * LC	0.0005	0.7416
ADP * LC	0.0001	0.0865
alanine * LC & NMR	0.366	0.0283
allantoin* LC & NMR	<0.0001	0.0001
alpha-aminobutyric acid * NMR	0.0027	0.0176
alpha-Lactose * LC	0.0567	0.0479
AMP * LC	0.04189	0.0069
androsterone	0.0012	0.0176
arginine * LC & NMR	<0.0001	0.0001
asparagine * LC & NMR	0.9081	0.0296
aspartate * LC & NMR	0.0002	0.0030
atenolol	0.0647	0.0409
betaine* LC & NMR	0.0433	0.0479
cAMP * LC	<0.0001	0.0001
carnitine * LC	0.0039	0.0001
CTP* LC	0.0002	0.0632
CDP* LC	0.0001	0.0332
CDP-choline/Citicoline * LC	<0.0001	0.0001
CDP-ethanolamine * LC	0.9081	0.0101
choline* LC & NMR	0.0130	0.0750
citrate * LC & NMR	0.5254	0.0205
citrulline * LC	0.5637	0.0176
creatine * LC	0.0001	0.4288
cystathionine * LC & NMR	0.0000	0.0001
cysteate * LC	0.0002	0.0001
cysteine * LC	0.0032	0.0101
cytidine * LC	0.0001	0.0015
deoxyadenosine * LC	0.0001	0.0001
D-fructose 6-phosphate * LC	0.0003	0.0004
dGDP * LC	0.0067	0.0210
D-galactose * LC	0.1842	0.1661
D-glucarate * LC	0.0018	0.0250
D-gluconic acid * LC	0.0003	0.0005
D-glucosamine * LC	0.0833	0.0024
D-glucosamine-6-phosphate * LC	<0.0001	0.0001
D-glucose * LC	<0.0001	0.0001
D-ribose * LC	0.2040	0.6924
dTMP * LC	0.0153	0.0001
dUMP * LC	<0.0001	0.0260
D-xylonate * LC	0.1190	0.0409
ethanolamine phosphate * LC	0.0015	0.0003
FMN * LC	0.0005	0.0250
fumarate * LC & NMR	0.4884	0.0349
gamma glutamyl ornithine * LC	0.3865	0.0030
GDP * LC	0.0567	0.0349
glucosamine * LC	0.0001	0.2353
glucose 6-phosphate * LC	<0.0001	0.1469

metabolites (10-day-adult)	*P* value *glp-1* VS N2	*P* value *daf-16;glp-1* VS N2
glycerophosphocholine * LC & NMR	0.0094	0.1294
glycine * LC & NMR	0.5254	0.0001
GMP * LC	0.0153	0.0008
guanine * LC	0.0001	0.0001
guanosine * LC	<0.0001	0.0001
hexadecenal * LC	0.0094	0.0001
hexanoylcarnitine * LC	<0.0001	0.0001
hippuric acid * LC	0.2987	0.0003
histamine * LC	0.0018	0.0037
histidine * LC	<0.0001	0.0001
homocarnosine * LC	<0.0001	0.0001
hypotaurine * LC	<0.0001	0.0001
hypoxanthine * LC	0.0327	0.0122
IMP * LC	0.0327	0.0016
indoleacetic acid * LC	0.6442	0.0004
inosine * LC	<0.0001	0.0001
isocitric acid * LC	0.0328	0.0018
isoleucine * LC & NMR	0.0001	0.0008
kynurenic acid * LC	<0.0001	0.2103
kynurenine * LC	0.0001	0.0001
leucine * LC & NMR	0.0111	0.0197
leukotriene E4 * LC	0.0153	0.0349
lysine * LC & NMR	<0.0001	0.2353
malate * LC & NMR	0.5637	0.0257
mannitol-1-phosphate * LC	0.3865	0.0993
mannobiose * LC	0.6861	0.5097
methionine * LC & NMR	0.0002	0.0001
N6-acetyl-L-lysine * LC	0.2253	0.0002
N-acetyl-D-glucosamine * LC	0.0153	0.0001
N-acetyl-L-glutamate * LC & NMR	<0.0001	0.0001
N-acetyl-L-leucine * LC	0.0567	0.0012
N-acetylmethionine * LC	0.0377	0.3913
N-acetylputrescine * LC	0.2987	0.0016
NAD+ * LC	0.0496	0.0001
NADH * LC	0.0002	0.0122
nicotinate * LC	0.4189	0.3913
octanoylcarnitine * LC	0.0039	0.0006
O-phosphoethanolamine * LC & NMR	0.0153	0.0010
ornithine * LC	0.0002	0.0001
oxaloacetate * LC	<0.0001	0.0072
palmitoylcarnitine * LC	0.0047	0.0046
phenylacetaldehyde * LC	0.0001	0.0016
phenylalanine * LC	0.0032	0.0001
phenylpyruvic acid * LC	<0.0001	0.0001
phosphorylcholine * LC & NMR	<0.0001	0.0069
proline * LC & NMR	<0.0001	0.0001
purine * LC	<0.0001	0.0001
pyrimidine * LC	0.0001	0.0001
pyroglutamic acid * LC & NMR	<0.0001	0.0001
s-adenosyl-L-homocysteine * LC	<0.0001	0.0012
serine * LC & NMR	0.0067	0.0001
sn-glycero-3-phosphoethanolamine * LC	0.0008	0.0024
spermidine * LC	<0.0001	0.0001
succinate * LC & NMR	0.0001	0.0497
taurine * LC	0.7728	0.0037
thiamine * LC	0.6033	0.0004
threonine * LC & NMR	<0.0001	0.0001
thymine * LC	0.0002	0.0001
trehalose * LC & NMR	0.2987	0.0001
tyramine * LC	<0.0001	0.0001
tyrosine * LC & NMR	0.0008	0.0003

**metabolites (10-day-adult)**	***P* value**<br>***glp-1* VS N2**	***P* value**<br>***daf-16;glp-1* VS N2**
UMP* LC	0.009	0.6442
uracil * LC	0.0002	0.7253
urate* LC	0.0433	0.0008
valine * LC & NMR	0.0209	0.0001
xanthine* LC	<0.0001	0.0001
xanthosine* LC	<0.0001	0.0001
xanthurenic acid* LC	0.0022	0.0101
oxidized glutathione * LC & NMR	0.0001	0.0001
